# Synergistic Cytotoxicity of Extracts of Chaga Mushroom and Microalgae against Mammalian Cancer Cells In Vitro

**DOI:** 10.1155/2024/7944378

**Published:** 2024-01-17

**Authors:** Sajeev Wagle, Julie Anne Lee, H. P. Vasantha Rupasinghe

**Affiliations:** ^1^Department of Plant, Food, and Environmental Sciences, Faculty of Agriculture, Dalhousie University, Truro NS B2N 5E3, Canada; ^2^Adored Beast Apothecary, 77 Rooney Crescent, Moncton NB E1E 4M4, Canada; ^3^Department of Pathology, Faculty of Medicine, Dalhousie University, Halifax NS B3H 4H7, Canada

## Abstract

Chaga mushroom (*Inonotus obliquus*) contains bioactive metabolites and has been used to treat various ailments, including cancer. Similarly, marine microalgae are considered a sustainable food supplement with anticancer and antioxidant properties. This study investigated the cytotoxicity of different extracts prepared from *I. obliquus* and microalgae using cultured human and canine cancer cell lines (MCF-7, HepG2, HOS, D-17, and DH-82). MTS cell viability assay was used to study the cytotoxicity of *I. obliquus* and microalgae extracts, and a synergy matrix effect was used to study the combined effect of the extracts. Isobologram analysis and the highest single agent synergy model were applied to study and validate the synergy between the extracts from *I. obliquus* and microalgae. Ethanol-based extraction and supercritical water extract significantly inhibited the growth of various mammalian cancer cells compared to aqueous extracts. Osteosarcoma cells were more susceptible to the supercritical extracts of *I. obliquus* and chlorophyll-free and sugar-free ethanol extracts of microalgae. A combination of ethanol-based *I. obliquus* extract and chlorophyll-free microalgae extract resulted in a synergistic interaction with various tested cancer cells. This study provides experimental evidence supporting the potential therapeutic application of *I. obliquus* and microalgae extracts with a synergistic effect to inhibit the growth of various mammalian cancer cells. Additional in vivo studies are required to fully explore possible therapeutic applications of these unique mixtures to be used in treating cancers.

## 1. Introduction

Natural compounds isolated from fungi and microalgae have various health benefits for humans, including in the treatment of various cancers [1–3]. The effectiveness of cancer treatments has significantly improved in the last 2 decades due to the advancement in multimodal imaging, immunotherapy, radiotherapy, neoadjuvant combinations, targeted therapies, and improved vaccines and biomarkers [4, 5]. However, cancer treatments have mostly relied on specific cytotoxic chemotherapy, and their impact on dividing healthy cells results in adverse side effects. There is a renewed interest in natural compounds that can inhibit the growth and metastasis of cancer, and investigations of their adjuvant effects with traditional chemo-drugs are underway [6, 7].

*Inonotus obliquus* is a parasitic fungus infecting hardwood trees mostly from the genus Betula (birches), which are commonly distributed in Russia, China, Japan, Korea, Eastern and Northern Europe, and Northern America [8]. *I. obliquus* is already considered a traditional medicine and has been used in the treatment of several human diseases, including cancer [9, 10]. Recent studies have shown the anticancer and anti-inflammatory potential of *I. obliquus* [10–12]. The fruiting body of *I. obliquus* produces a diverse range of natural compounds, such as flavonoids, triterpenoids, alkaloids, and inositol. Recently identified triterpenoid lanosterols have elicited interest because of their potent in vitro cytotoxicity in mammalian cells, regardless of p53 mutation status [10, 11, 13].

Unicellular marine microalgae-derived compounds have shown anticancer, antimicrobial, anti-inflammatory, and immunomodulatory properties [14, 15]. Among microalgae, *Tetraselmis chuii* has received increased interest as a sustainable source of supplemented food with low-carbon/nitrogen footprints and naturally healthy contents, including amino acids, essential fatty acids, vitamins B, C, and E, and dietary antioxidants [16]. *T. chuii* had the highest (poly)phenol content and DPPH radical scavenging activity among the tested species of microalgae [17]. Similarly, a lipid-rich extract of *Nannochloropsis gaditana* has shown a selective cytotoxicity effect on colon carcinoma HCT-116 cells but not on nontumorigenic cells [18]. Methanol extracts of *Chlorella vulgaris* exhibited cytotoxicity against human prostate cancer (PC-3), hepatocellular carcinoma (HEPG-2), colorectal carcinoma (HCT-116), and epithelioid carcinoma (Hela) cells in vitro [19]. Supplementation of diet with microalgae also improved hematological and biochemical parameters in athletes [20].

The combination of two different drugs has been a new trend to treat human ailments, as it improves drug efficacy by reducing the dose and limiting toxicity. This combination therapy has been shown to be effective in overcoming the chemoresistance problem in the treatment of cancer [21, 22]. Medicinal plant-derived phytochemical compounds have been identified and characterized for low cytotoxicity compared to synthetic chemotherapeutic drugs. In general, phytochemicals significantly induce apoptosis, suppress angiogenesis, and modulate the activity of enzymes to inhibit the metastasis of cancer [23, 24]. Thus, this study aims to understand the combined effect of extracts from *I. obliquus* and microalgae using human and canine cancer cell lines with the ultimate goal of developing potential natural health products (NHP) for the treatment of mammalian cancers.

## 2. Materials and Methods

### 2.1. Preparation of Five Extracts of *I. obliquus*

**Figure 1:**
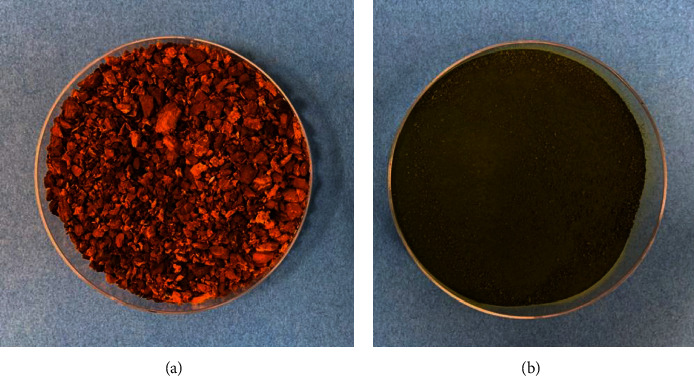
Dehydrated Chaga mushroom *I. obliquus* (a) and microalgae (b) materials were used for the extraction of natural compounds in this study.

Five different extracts from *I. obliquus*, namely MH-E1 (ethanol), MH-H_2_O (hot water), MH-E1-SF (sugar-free), MH-SFE1b (supercritical high pressure), and MH-SFE1c (supercritical with ethanol as cosolvent), were used for cytotoxicity analyses (Table 1). Raw materials of *I. obliquus* (Figure 1(a)) that were used in this study were provided by Adored Beast Apothecary, Moncton, NB, Canada.

To prepare MH-E1, 10 g of dry and finely ground *I. obliquus* powder was dissolved in 200 mL of 100% ethanol (1 : 20 solid/solvent ratio), sonicated for 20 min twice at room temperature, centrifuged (3,000x *g* for 10 min), and filtered through P8 filters (Fisher Scientific, Ottawa, ON, Canada). The filtrate was collected and bioactives were re-extracted using 100 mL of 100% ethanol. The combined filtrates were concentrated by rotary vacuum evaporation and the concentrated extracts were freeze-dried to obtain a dry extract.

For sugar-free *I. obliquus* extract (MH-E1-SF) preparation, 30 g of *I. obliquus* powder was dissolved in 600 mL of 100% ethanol and sonicated for 20 min twice at room temperature, centrifuged (3,000x *g* for 10 min), and filtered (P8 filters). The residues were re-extracted using an additional 300 mL of 100% ethanol. Both filtrates from the first and second extracts were combined and concentrated (about 200 mL) using rotary vacuum evaporation. A flash chromatography column (6.5 cm × 45 cm, Sati International Scientific Inc., Dorval, QC, Canada) was packed with 400 g of sorbent beads (Sorbent SP207-05 Sepabeads resin brominated styrenic adsorbent; 0.25 mm diameter, 630 m^2^/g surface area; Sorbent Technologies, Atlanta, GA, USA). After the column was equilibrated overnight using 50% aqueous ethanol, the concentrated extract was mixed with deionized (DI) water (1/1 v:v) and loaded into the chromatography column. Sugars in the loaded extracts were eluted with DI water while monitoring the Brix value to reach below 0.1% using a digital refractometer (Model 300016, Sper Scientific, Scottsdale, AZ, USA). Then, the bound bioactives were eluted using 2 L of 95% ethanol. The ethanol elutes were concentrated by rotary vacuum evaporation and the concentrate was freeze-dried.

To prepare a hot water extract (MH-H_2_O), 25 g of *I. obliquus* powder was mixed with 500 mL of DI water and heated at 80 ± 2°C for 2 hr in a shaking water bath (60 rpm). The sample was centrifuged at 3,000x *g* for 15 min and filtered with vacuum-aided P8 filters, followed by freeze-drying to obtain a dry powder.

Two supercritical extracts MH-SFE1b and MH-SFE1c were prepared using the supercritical water extraction process (Diversified Metal Engineering Ltd., Charlottetown, PE, Canada). Forty grams of dried and finely ground *I. obliquus* powder were processed with a pressure of 9,000 psi (for MH-SFE1b) and 7,500 psi (for MH-SFE1c), a temperature of 50°C, and an extraction time of 1 hr. A cosolvent of 10% ethanol was used for the MH-SFE1c supercritical fluid extract. The resulting extracts were freeze-dried.

### 2.2. Preparation of Three Extracts of Microalgae

The source of microalgae comprises 60% w/w *N. gaditana*, 15% w/w *T. chuii*, and 25% w/w *C. vulgaris* (Figure 1(b)) that were provided by Adored Beast Apothecary, Moncton, NB, Canada. Three extracts of microalgae, i.e., GLE1 (ethanol), GLE1-CF (chlorophyll-free), and GLE1-CF-SF (chlorophyll- and sugar-free), were used for cytotoxicity analyses (Table 1). To prepare the GLE1 extract, 30 g of dry microalgae mixture was dissolved in 600 mL of 100% ethanol (1 : 20 solid/solvent ratio), sonicated for 20 min twice at room temperature, centrifuged (3,000x *g* for 10 min), and filtered through P8 filters (Fisher Scientific, Ottawa, ON, Canada). The filtrate was collected, and bioactive compounds were re-extracted using 300 mL of 100% ethanol. Filtrates were combined and concentrated by rotary vacuum evaporation, and the concentrated extracts were freeze-dried to produce the dry extract.

To prepare the chlorophyll-free microalgae extract (GLE-CF), first, 2.5 g of microalgae mixture was added to 900 mL of extraction solvent (530 mL of ethanol, 300 mL of DI water, and 70 mL of acetonitrile) and sonicated for 20 min twice at room temperature. This combination of solvents provided the best separation of chlorophyll fraction in the next step. The sample was centrifuged at 3,000x *g* for 10 min and filtered through P8 filters. The filtrate was collected into a separatory funnel, and 100 mL *n*-hexane was added, which was then vigorously shaken while degassing. The chlorophyll-free aqueous phase was collected and concentrated by rotary vacuum evaporation, followed by freeze-drying to generate a dry powder of GLE1-CF.

For the preparation of the chlorophyll- and sugar-free extract of microalgae (GLE1-CF-SF), a concentrated sample of GLE1-CF was mixed in absolute ethanol (1 : 1 v/v) and loaded into a flash chromatography column as explained above for MH-E1-SF. Briefly, the sugars were eluted with DI water until the Brix value of eluting fell below 0.1 (as determined with a digital refractometer). The bound bioactives to the stationary phase of the column were eluted with 2 L of absolute ethanol. The elute was concentrated by rotary vacuum evaporation and the concentrated extracts were freeze-dried to produce the dry extract. A stock solution of 50–100 mg/mL was made in dimethyl sulfoxide (DMSO), filtered using a 0.22 *µ*m syringe filter, aliquoted, and stored at −20°C.

### 2.3. Quantification of Major Phytochemicals of the Extracts by Ultra-Performance Liquid Chromatography Coupled with Electrospray Ionization and Mass Spectrometry (UPLC-ESI-MS)

The standard stock solutions of extracts (1,000–10,000 *µ*g/mL) were prepared by accurately weighing the dried extracts and dissolving them in methanol. Ultra-high performance liquid chromatography (UPLC, Model H-class system, Waters, Milford, MA, USA) was used for analyzing the extracts. For the analysis of isoprenoids, an xBridge™ phenyl column (4.6 × 100 mm, 5 *μ*m) (Waters, Milford, MA, USA) was used with a gradient elution carried out with 0.1% formic acid in water (solvent A) and 0.1% formic acid in acetonitrile (solvent B), with a flow rate of 0.4 mL/min and an injection volume of 2.0 *μ*L. A linear gradient profile was used with the following proportions of solvent A applied at time *t* (min); (*t*, *A*%): (0, 90%), (1, 90%), (7, 50%), (16, 0%), (18, 0%), (20, 90%). For the analysis of (poly)phenols, an acquity UPLC BEH C18 column (2.1 mm × 100 mm, 1.7 *μ*m) (Waters, Milford, MA, USA) was used with gradient elution, carried out with 0.1% formic acid in water (solvent A) and 0.1% formic acid in acetonitrile (solvent B), with a flow rate of 0.2 mL/min and an injection volume of 2.0 *μ*L. A linear gradient profile was used with the following proportions of solvent A applied at time *t* (min); (*t*, *A*%): (0, 94%), (2, 83.5%), (2.61, 83%), (2.17, 82.5%), (3.63, 82.5%), (4.08, 81.5%), (4.76, 80%), (6.75, 20%), (8.75, 94%), and (12, 94%).

MS analysis was performed with a Micromass Quattro micro API MS/MS system, controlled by the MassLynx V4.1 data analysis system (Micromass, Cary, NC, USA), as described by Rupasinghe et al. [25]. Electrospray ionization in negative ion mode (ESI−) was used for the ionization of all the analytes. The mass spectrometry conditions included a capillary voltage of 3,000 V with nebulizing gas (N2) at a temperature of 375°C. The cone voltage (25–50 V) was optimized for each compound. The analytes were identified using single ion monitoring (SIM) mode and quantified using calibration curves generated using external standards. The SIM mode used (*m/z*) and retention time (RT) of each analyte are given in Table 2.

### 2.4. Cell Lines, Culture Conditions, and Reagents

The following mammalian cell lines were purchased from ATCC through Cedarlane, Burlington, ON, Canada: MCF-7 (ATCC-HTB-22, human breast epithelial adenocarcinoma), HepG2 (ATCC-HB-8065, human hepatocellular carcinoma), HOS (ATCC-CRL-1543, Human Osteosarcoma), D-17 (ATCC-CCL-183, Canine osteosarcoma), DH-82 (ATCC-CRL-10389, canine histiocytosis), MCF10-A (ATCC-CRL-10317, human breast epithelial cell), AML-12 (ATCC-CRL-2254, mice liver epithelial cell), THLE-3 (ATCC-CRL-11233, human liver epithelial), and CnOb (cell applications-canine osteoblast). DH-82, HOS, and D-17 cells were cultured in EMEM (Sigma–Aldrich, Oakville, ON, Canada), MCF-7 and HepG2 in DMEM (Gibco), CnOb in CnOb basal medium supplemented with growth medium (cell applications), MCF-10A cultured in basal medium supplemented with growth medium, and THLE-3 in BEGM with supplements (Lonza Bioscience, Burlington, ON, Canada), as recommended. The cells were supplemented with 15% fetal bovine serum as required (Fisher Scientific, Ottawa, ON, Canada) and antibiotic (100 U/mL penicillin and 100 *µ*g/mL streptomycin). The cells were maintained in a 5% CO_2_ humidified incubator at 37°C (Fisher Scientific, Ottawa, ON, Canada). All experiments were performed after the second passage of the cells and repeated at least three times, independently.

### 2.5. Cell Viability Assay

Crude ethanol- and water-based extracts, a sugar-free extract, supercritical extracts from the *I. obliquus* group, and ethanol-based chlorophyll- and sugar-free extracts from the microalgae group were selected for cell viability assays. IC_50_ values of the extracts in each cell line were confirmed with MTS colorimetric assay [26]. Briefly, the cells (5,000–10,000 cells/well) were seeded into 96-well plates, incubated overnight, and then treated with either DMSO control, 1, 50, 100, 300, or 500 *µ*g/mL of each extract for 24 hr. Next, MTS/PMS reagent (Promega, Madison, WI, USA) was added and incubated 2–3 hr in the humidified CO_2_ incubator. Absorbance was measured at 490 nm (Infinite™ 200 series, Tecan, Männedorf, Switzerland). Background absorbance from the culture medium, DMSO, and extracts was subtracted to estimate the viability percentage. GraphPad Prism V 8.0 (GraphPad Software Inc., San Diego, CA, USA) was used to calculate IC50 by the sigmoidal dose–response curve.

### 2.6. Combination Treatment and Determination of Combination Index

With the IC_50_ scores from the MTS viability assay, MH-E1-SF from the *I. obliquus* group and GLE1-CF-SF from the microalgae group were selected for the determination of synergistic effects, as both extracts showed strong significance in lower concentrations when compared with the other extracts. A viability and synergy matrix of the two extracts with concentrations of 0, 25, 50, 100, 150, and 200 *µ*g/mL in a ratio of 1 : 1, 1 : 2, and 1 : 4 in a 96-well plate was assayed after 24 hr. The combination effect between the two selected ex-tracts was quantified using the method of isoboles [27], and a dose–response surface was mapped using the highest single agent (HAS) model of synergy using Combenefit software [28]. The isoboles method uses the IC_50_ doses of individual drugs as intercept values in which doses are represented on *x*- and *y*-axes, represented by a simple linear equation of a/A + b/B = 1, where a is the dose of drug A and b is the dose of drug B when the two drugs are used in combination. The Chou–Talalay method was applied to determine the combination index (CI) in the analysis of the combination study [29, 30], which is represented by CI = ((D)1/(Dx)1) + ((D)2/(Dx)2), where (Dx)1 and (Dx)2 are the EC_50_ doses of drugs 1 and 2 alone, respectively, that give the specified effect, and (D)1 plus (D)2 is the combination dose that produces this effect. The effect of the combination of the two drugs was confirmed from the CI values, where CI < 1 demonstrates synergism, CI = 1 indicates additive effect, and CI > 1 indicates antagonism.

The HSA response surface model was also generated using Combenefit software (Cambridge, UK) to map the synergy. The HSA synergy model states that the expected combination effect equals the higher effect of individual drugs. Therefore, any additional effect over the higher single drug will be considered as an HSA synergy [31]. In the color map, light to dark blue shows increased synergy while yellow–red shows an antagonistic effect on the Combenefit mapped surface HAS plot and matrix plot.

### 2.7. Statistical Analysis

All the results are expressed as the mean of triplicate experiments, and values are expressed as mean ± standard deviation (SD). Analysis of variance with post hoc Tukey and Sidak's test was used for multiple comparisons using GraphPad Prism 8. Analysis and data visualization of the synergy of drug combinations were performed using Microsoft Excel, Combenefit software, and GraphPad Prism 8. A value of *p* < 0.05 was considered statistically significant.  ^*∗*^*p* < 0.05,  ^*∗∗*^*p* < 0.01, and  ^*∗∗∗*^*p* < 0.001.

## 3. Results

### 3.1. Quantification of Major Metabolites of *I. obliquus* and Microalgae Bioactive Using UPLC-ESI-MS

Extracts of *I. obliquus* and microalgae were analyzed by UPLC-ESI-MS using the SIM mode and RT of the external standards. Triterpenes (betulinic acid and trametenolic acid; only in *I. obliquus* extracts), phenolic acids (*p*-hydroxy benzoic acid, cinnamic acid, dihydroferulic acid, sinapic acid, protocatechuic acid, caffeic acid, and syringic acid), and flavonoids (taxifolin, catechin, and quercetin) were present in the extracts, and their concentration varied depending on the source and extraction method (Table 2). The presence of triterpenes and various (poly)phenols has already been screened, and these compounds have been verified as anticancer agents in previous studies. The UPLC-ESI-MS analysis in our study validated that the major bioactives of *I. obliquus* and microalgae had been recovered and concentrated via the extraction methods used. Aqueous extracts contained high concentrations of (poly)phenols, whereas ethanol-based extracts had high concentrations of triterpenes. The trametenolic acid content was significantly high in the supercritical fluid extract.

### 3.2. Cytotoxic Effects of *I. obliquus* Extracts on Mammalian Cancer Cells

**Figure 2:**
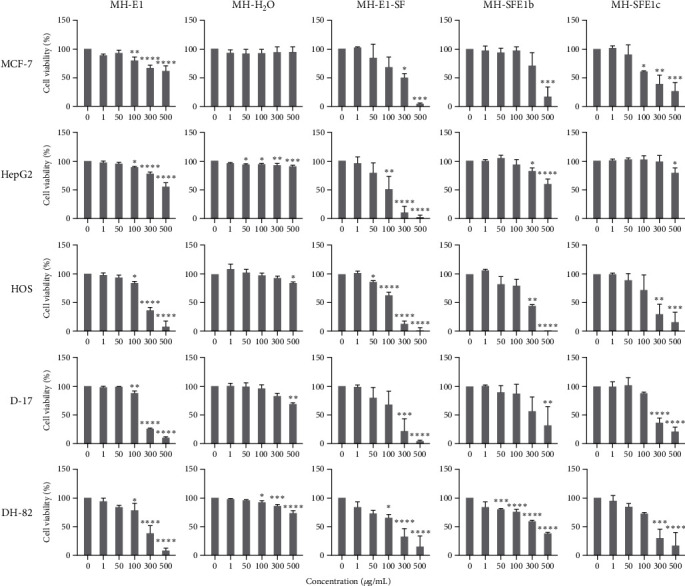
Effects of different *I. obliquus* extracts in mammalian cancer cell lines. Cells were treated with different extracts (see Table 1) and doses of *I. obliquus*, and cell viability was measured using the MTS assay. Each graph represents mean viability expressed in percentage of control. One-way ANOVA was used to compare the dose-dependent toxicity.  ^*∗*^*p* < 0.05,  ^*∗∗*^*p* < 0.01,  ^*∗∗∗*^*p* < 0.001, and  ^*∗∗∗∗*^*p* < 0.0001, compared with control group. MCF-7, human breast epithelial adenocarcinoma; HepG2, human hepatocellular carcinoma; HOS, human osteosarcoma; D-17, canine osteosarcoma; and DH-82, canine histiocytosis.

*I. obliquus* extracts were assessed for dose-dependent cytotoxicity using various mammalian cancer cells (Figure 2 and Table 3). Compared to other extracts, the IC_50_ of the water-based extracts (MH-H_2_O) was very high for all the tested cell lines (≥1,000 *µ*g/mL). Sugar-free ethanol extracts showed a strong cytotoxic effect on the cancer cells with lower IC_50_ values (115–187 *µ*g/mL) compared to the ethanol extracts (208–769) (Table 3). Ultrasonication-assisted ethanolic extracts of *I. obliquus* inhibited the growth of human and canine sarcoma cancer cell lines significantly with 100 *µ*g/mL concentration, while sugar-free ethanol extracts significantly inhibited proliferation with as low as 50 *µ*g/mL in HOS cells and 100 *µ*g/mL in HepG2 and DH-82 cells and 300 *µ*g/mL in the MCF-7 and D-17 cell lines. MCF cells and D-17 cells were relatively resistant to lower doses of the extracts. Similarly, supercritical extracts were mostly effective with higher doses in various cancer cell lines except for HepG2. HepG2 cells were relatively resistant to supercritical extracts, and only higher doses (>300 *µ*g/mL) inhibited cell proliferation. Supercritical extracts MH-SFE1b and MH-SFE1c inhibited cancer cell proliferation with doses of 300 *µ*g/mL. However, DH-82 cells were more susceptible to supercritical extract MH-SFE1b, with the lowest effective dose of 50 *µ*g/mL. DH-82 cells showed stronger cytotoxicity, even with the water extracts and supercritical extracts of MH-SFE1b (50 *µ*g/mL), and with concentrations as low as 100 *µ*g/mL of ethanol extracts.

### 3.3. Cytotoxic Effects of Microalgae Extracts on Mammalian Cancer Cells

**Figure 3:**
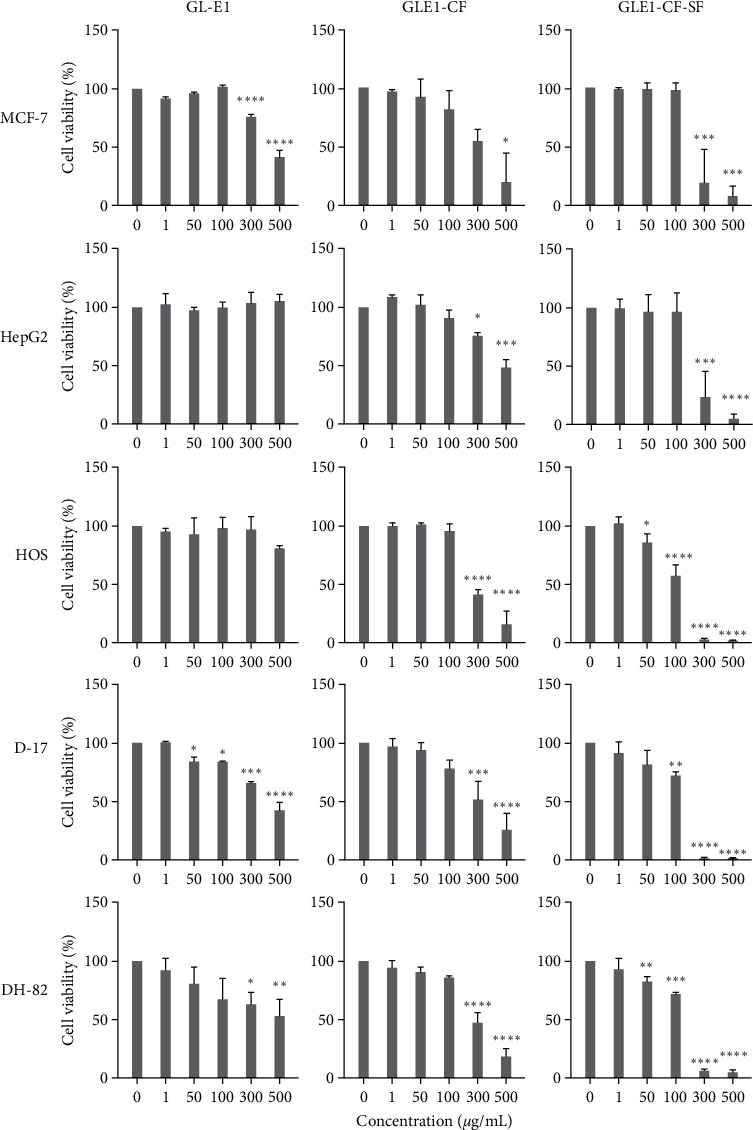
Effect of microalgae extracts in mammalian cancer cells. Cancer cells were treated with different extracts (see Table 1) of microalgae, and cell viability was measured after 24 hr using MTS assay. Each graph represents mean viability expressed in percentage of control. One-way ANOVA was used to compare the dose-dependent toxicity.  ^*∗*^*p* < 0.05,  ^*∗∗*^*p* < 0.01,  ^*∗∗∗*^*p* < 0.001, and  ^*∗∗∗∗*^*p* < 0.0001, compared with control group. MCF-7, human breast epithelial adenocarcinoma; HepG2, human hepatocellular carcinoma; HOS, human osteosarcoma; D-17, canine osteosarcoma; and DH-82, canine histiocytosis.

Ethanol, chlorophyll-free, and chlorophyll- and sugar-free extracts were assessed for their cytotoxic effects against the MCF-7, HepG2, HOS, D-17, and DH-82 cancer cell lines (Figure 3 and Table 3). The five selected cancer cell lines represent common cancer types in both humans and canines. HepG2 cells were resistant to the GLE1 extract, while doses greater than 300 *µ*g/mL were toxic to most of the cancer cells. All the cancer cells were more susceptible to the GLE-CF-SF extract compared to GLE1 and GLE1-CF. IC_50_ doses were reduced significantly in HOS, D-17, and DH-82 cell lines by removing the sugar of the extract. D-17 and DH-82 canine osteosarcoma and histiocytic sarcoma were susceptible to both ethanol, sugar-free, and/or chlorophyll extracts; however, HOS human osteosarcoma cells were not affected by ethanol extracts, although 50 *µ*g/mL doses of sugar- and chlorophyll-free extracts significantly inhibited the cell proliferation.

### 3.4. Supercritical Extracts of *I. obliquus* Selectively Inhibited Breast and Osteosarcoma Cell Viability

**Figure 4:**
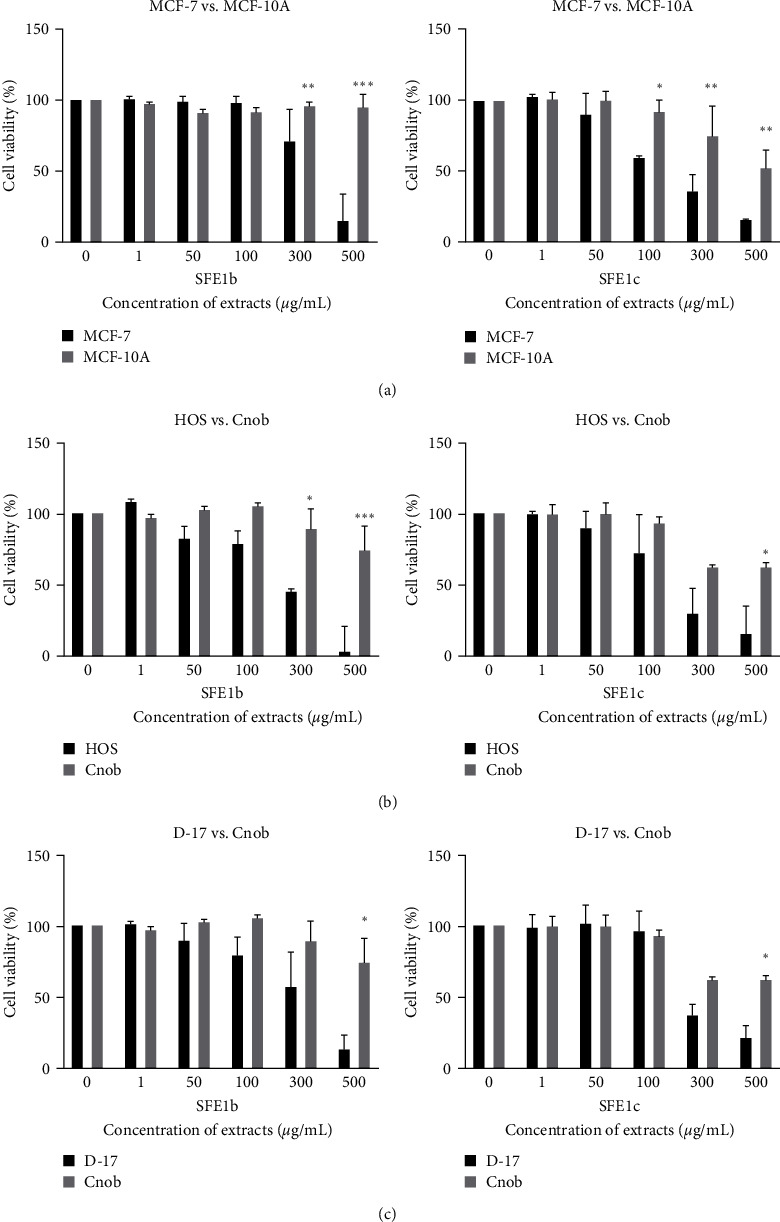
Effect of supercritical *I. obliquus* extracts on cancer and normal mammalian cells. Cancer cell and nonmalignant normal cell lines were treated with various doses of two supercritical extracts (see Table 1) of *I. obliquus*, and cell viability was determined after 24 hr. (a) Comparison between human breast epithelial adenocarcinoma (MCF-7) and human breast epithelial cell (MCF-10A) cells. (b) Comparison between human osteosarcoma (HOS) and canine osteoplast (Cnob) cells. (c) Comparison between canine osteosarcoma (D-17) and Cnob cells.  ^*∗*^*p* < 0.05,  ^*∗∗*^*p* < 0.01, and  ^*∗∗∗*^*p* < 0.001.

Two supercritical extracts MH-SFE1b and MH-SFE1c were assessed against cancer cell lines and healthy, nonmalignant cell lines (Figure 4). Interestingly, we found that these supercritical extracts were selectively cytotoxic to the MCF-7 breast cancer cell line, human osteosarcoma (HOS), and canine osteosarcoma (D-17), while sparing the corresponding healthy cell lines, i.e., MCF-10A, human breast epithelial cell and canine osteoblast (CnOb). Both breast epithelial cells and canine osteoblast cells exhibited significantly higher viability at 300 and 500 *µ*g/uL of the extracts than that of breast cancer and osteosarcoma cells (Figure 4). Interestingly, MCF-10A cell proliferation was not affected, even at higher concentrations of 300 and 500 *µ*g/mL of the extracts, while the same doses were cytotoxic to cancer cell line MCF-7. Similarly, CnOb cells showed above 70% viability at 500 *µ*g/mL of MH-SFE1b, while the same concentration of the extract was highly toxic in human and canine osteosarcoma cells, i.e., HOS and D-17. However, murine hepatocyte cells (AML-12), liver epithelial cells (THLE-3), and RAW 264.7 macrophage cell viability were inhibited by supercritical extracts when compared with cancer cells HepG2 and DH-82 with the same drug dose (data not available). Moreover, other extracts used in this study did not show selective cytotoxicity to tested cancer cell lines (data not available). These preliminary results suggest that supercritical extracts of *I. obliquus* are selectively cytotoxic to cancer cell lines in vitro.

## 4. Synergistic Effect of I. obliquus and Microalgae Extracts against Mammalian Cancer Cells

**Figure 5:**
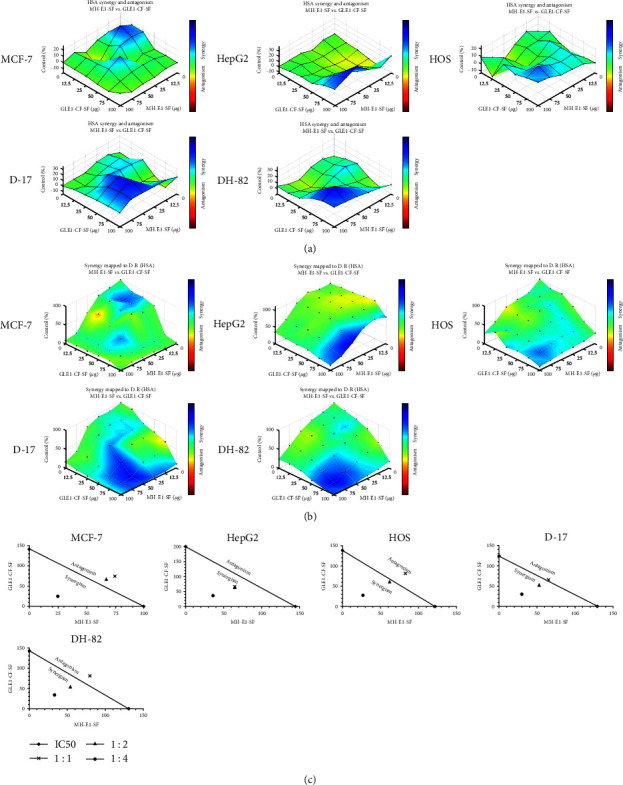
Synergistic effects of *I. obliquus* and microalgae extracts on mammalian cancer cells. Cancer cells were treated with MH-E1-SF or GLE1-CF-SF extracts (see Table 1) alone and in combination for 24 hr. (a) Synergy and antagonism space, as mapped by the highest single agent (HSA) model of synergy, expressed as a percentage of control. The level of synergism (blue) or antagonism (red) at each concentration is represented by color scale bar. (b) Experimental two extract combination dose–response surface, expressed as a percentage of the control value. The level of synergism (blue) or antagonism (red) at each concentration is represented by the color scale bar. All experiments were conducted at least three times. (c) The synergy of each combination was assessed using the combination index from the isobologram analysis (Loewe model of synergy). A combination index with less than 1 shows synergy, equal to 1 shows an additive effect, and greater than 1 shows an antagonistic effect.

In comparison to all the tested extracts, MH-E1-SF (MH) and GLE1-CF-SF (GL) had the lowest IC_50_ in all the cell lines. Thus, these two extracts were selected for the determination of the synergistic effect using various combinations of the extracts (Table 4). MH and GL extracts were treated in a synergy matrix alone or combination in 96-well plates. Cancer cells responded with a dose-dependent effect to the different tested combinations (Figure 5). An analysis of the CI based on the Loewe additivity model can be visualized in isobologram. The combination of MH and GL resulted in an additive effect (combination values of two drugs in a straight line) with a 1 : 1 combination in MCF-7, HOS, D-17, and DH-82 cells and a synergistic effect (below the straight line) in HepG2 cells. Similarly, the combination of 1 : 2 and 2 : 1 MH and GL had an additive effect in MCF-7 cells and a synergistic effect in other cell lines. Additionally, the combination of 1 : 4 and 4 : 1 MH and GL resulted in a synergistic effect in all the tested cell lines (Figure 4(c) and Table 4). Similarly, the HSA model of drug synergy showed strong synergism between MH and GL extracts. The data obtained from three biological replicates mapped in 3D graphs show the percentage of synergism (blue to dark blue) with different combination ratios between the extracts (Figures 5(a) and 5(b)). Each point represents the effective concentration (EC_50_) of the different combinations of MH and GL, whereby height above zero in the third dimension indicates synergism. If the coordinates of the drug doses are located on the plane, it represents additivity. Most of the combinations mapped by the HAS model and isobolograms showed strong synergism in osteosarcoma and histiocytic sarcoma cells.

## 5. Discussion

Cancer is a condition whereby cells grow abnormally, invade the surrounding environment, and metastasize to other organs of the body. Several risk factors are associated with cancer initiation and development [32]. Despite cancer treatment approaches continuously being improved and tailored to meet each patient's needs, a greater number of cancer cases are diagnosed every year. The age-standard cancer mortality rate has decreased in many cancers with newer drugs such as immunotherapy. However, the potential to advance primary treatment exists through combination therapy and adjuvant and neoadjuvant additives [5]. Chemotherapy has been an advantage for metastatic cancer treatment and increased survival of the patient; however, severe side effects, drug resistance, and relapse remain challenges [1]. As a result, treatment methods using phytochemicals as supplements or in combination with chemo-drugs are being studied. These combinations have significantly reduced tumor growth and metastatic potential; 25% of all newly approved anticancer drugs from 1981 to 2019 are derived from natural products [27, 33]. Various terpenoids and (poly)phenols such as flavonoids, catechin, and quinones have been reported to have an anticancer effect in vitro, and mouse studies and phytochemicals such as Vinca alkaloids, taxanes (docetaxel, paclitaxel), podophyllotoxin (etoposide, teniposide), and camptothecin are currently being used to treat cancers, i.e., nonsmall-cell lung carcinoma (NSCLC), osteosarcoma, breast, prostate, gastric carcinoma, ovarian, colorectal, and testicular carcinoma [33–36].

Several studies on *I. obliquus* extracts for their anti-inflammatory and anticancer properties have highlighted gallic acid, protocatechuic acid, caffeic acid, syringic acid, betulinic acid, syringic acid, and various triterpenoids including inotodiol and lanosterol as the main bioactive metabolites [37–39]. UPLC-ESI-MS characterization of the extracts showed a strong presence of betulinic acid and trametenolic acid, a moderate amount of phenolic acids such as ferulic acid, cinnamic acid, and syringic acid, and a low amount of cinnamic acid, sinapic acid, catechin, and quercetin. Most of these phytochemicals are recognized for their strong anticancer properties. Triterpenes in *I. obliquus* are being extensively studied for their anticancer properties in various mammalian cancer cells and mouse models [11, 40]. Inotodiol, trametenolic acid, 3b-hydroxylanosta-8,24-dien-21-al, and betulin are frequently identified in *I. obliquus* [9, 10, 41]. Several extracts from *I. obliquus* have shown cytotoxicity to various mammalian cancer cell lines, inducing cell cycle arrest and apoptosis signaling [10, 41–43]. Moreover, *I. obliquus* busone A has been identified as an important cytotoxic triterpenoid against human lung cancer cells [10]. Additionally, lanostane-type triterpenoid was reported to have a cytotoxic effect in murine leukemia cells (via caspase 3 dependent pathway) human lung adenocarcinoma cells and human bronchial epithelial cells [9, 11, 44].

Similar to previous findings, the ethanol and supercritical extracts of *I. obliquus* responded dose-dependently to human breast carcinoma, hepatocarcinoma, osteosarcoma, canine osteosarcoma, and histiocytic sarcoma cells [10]. Ethanol-based sugar-free extracts of *I. obliquus* were cytotoxic to all the cancer cell lines with lower IC_50_ values. HepG2 liver carcinoma cells were relatively resistant to *I. obliquus* supercritical extracts compared to other extracts. We tested the cytotoxicity of *I. obliquus* extracts on human osteosarcoma cells (HOS), canine osteosarcoma (D-17), and canine histiocytic sarcoma (DH-82) for the first time, and we found that the cellular cytotoxicity with supercritical fluid extracts of *I. obliquus* was relatively lower in breast epithelial cells and canine osteoblast as compared to human breast cancer cells and osteosarcoma cells. The supercritical extracts showed a significantly greater concentration of triterpene trametenolic acid. Further characterization of supercritical fluid extracts and their role in selective cytotoxicity is to be investigated.

Marine microalgae have already become an important source of dietary fiber, which has great health benefits, including proven antioxidant activities [17, 45]. They are rich in ascorbic acids, glutathione, tocopherols, poly-unsaturated fatty acids, and (poly)phenols. Recent studies have also focused on the potential health benefits of microalgae, including in the management of high blood pressure, type 2 diabetes, nonalcoholic fatty liver disease, obesity, inflammatory diseases, and certain cancers [14, 45, 46]. Our study also showed that the removal of chlorophyll and sugars from crude ethanol extracts could significantly improve the anticancer properties of microalgae. This is the first report on the cytotoxicity of extracts of microalgae in HOS, D-17, and DH-82 cell lines.

The combination of two extracts significantly reduced the effective drug dose in five of the cancer cell lines tested, as mapped by synergistic response surface analysis and isobolograms. The combination effect with ethanol-based sugar-free extracts of *I. obliquus* and chlorophyll- and sugar-free extracts of microalgae resulted in a synergistic reduction of cell viability in the selected cancer cells. As several studies are seeking to discover new drugs and reduce the doses of chemo drugs used in cancer treatments, the effect of these two extracts in combination has the potential for use as a natural anticancer therapeutic. Moreover, these extracts could have the potential to sensitize the effect of chemotherapeutic drugs, which would significantly improve patient health. However, one of the limitations of synergy maps is possible “overfitting.” Therefore, the in vivo efficacy of these extracts should be further studied using experimental animal models to elucidate the therapeutic efficacy and mechanism(s) of action.

## 6. Conclusions

In our search for alternative natural health products for mammalian cancers, we found that the cytotoxicity of *I. obliquus* and microalgae extracts in various human and canine cell lines exhibited strong anticancer activities. In conclusion, osteosarcoma cells were more susceptible to the supercritical extracts of *I. obliquus* and chlorophyll-free and sugar-free ethanol extracts of microalgae. Moreover, the combined effect of these extracts resulted in synergistic and additive drug dose effects. The phytochemical constituents of these extracts have been studied for their anticancer effect, focusing on their pharmacological and molecular mechanisms. The preliminary analysis has demonstrated the triterpenoid and phenolic acid classes of metabolites have contributed to the observed cytotoxicity activity. Taken together, the combinations of extracts of *I. obliquus* and microalgae exhibited potential for developing natural cytotoxic agents against various cancer cells. This strong cytotoxicity and drug synergism should be further investigated to validate their therapeutic applications clinically.

## Figures and Tables

**Table 1:** Extraction methods and total yield of the extracts of *I. obliquus* and microalgae.

Extracts	Extraction method	Starting quantity (g)	Final yield (mg)	Yield (%)
*I. obliquus*				
MH-E1	Ethanol (ultrasonication)	10	336	3.4
MH-E1-SF	Ethanol sugar-free (chromatography)	30	804	2.68
MH-H_2_O	Hot water	25	3,700	14.8
MH-SFE1b	Supercritical (9,000 psi, 50°C, 1 hr)	40	41	0.1
MH-SFE1c	Supercritical (7,500 psi, 50°C, 10% ethanol, 1 hr)	40	237	0.59
*Microalgae*				
GLE1	Ethanol (ultrasonication)	30	1,442	4.8
GLE1-CF	Ethanol (ultrasonication; chlorophyll-free)	2.5	270	10.8
GLE1-CF-SF	Ethanol (ultrasonication; chlorophyll- and sugar-free)	2.5	176.6	7.06

**Table 2:** Concentrations of major metabolites of various extracts of *I. obliquus* and microalgae.

Compound	*m/z*	RT (min)	Concentration (mg/g extract)							
			MH-E1	MH-H_2_O	MH-E1-SF	MH-SFE1b	MH-SFE1c	GL-E1	GLE1-CF	GLE1-CF-SF
Betulinic acid	455.2	3.57	2.99	0.01	1.56	15.2	1.21	ND	ND	ND
Trametenolic acid	455.2	3.67	37.49	0.26	21.46	334.4	17.48	ND	ND	ND
*p*-Hydroxy benzoic acid	137.0	3.9	7.32	2.63	1.81	0.57	6.84	0.09	0.07	0.80
Cinnamic acid	146.9	7.4	0.00	0.35	0.23	0.00	0.00	0.00	0.17	0.00
Dihydro ferulic acid	195.0	8.25	0.59	8.33	0.95	0.91	0.55	0.66	0.41	5.90
Sinapic acid	223.0	5.4	0.13	0.14	0.01	0.01	0.04	0.00	0.00	0.01
Protocatechuic acid	152.8	2.5	0.86	0.30	0.36	0.00	0.13	0.00	0.08	0.03
Caffeic acid	179.0	4.3	0.08	0.03	0.02	0.00	0.12	0.00	0.00	0.01
Syringic acid	197.0	3.9	13.98	109.8	11.60	0.03	6.31	0.03	0.27	0.15
Taxifolin	303.0	0.95	0.08	0.17	0.02	0.03	0.09	0.32	0.01	0.36
Catechin	288.7	3.4	0.42	9.16	3.72	0.00	0.00	0.00	0.00	0.00
Quercetin	300.7	6.8	0.57	1.67	0.39	0.00	0.00	0.00	0.00	0.00

ND, not detected; m/z, mass/charge used for the single ion monitoring mode; and RT, retention time. For the abbreviations of the extracts, please refer to Table 1.

**Table 3:** IC_50_ values (*µ*g/mL) of various extracts of *I. Obliquus* and microalgae against mammalian cancer cell lines.

Extracts	Mammalian cell lines				
	MCF7	HepG2	HOS	D-17	DH-82
*I. obliquus*					
MH-E1	769 ± 166	540 ± 7.9	219 ± 37.2	208 ± 8.6	196 ± 24.5
MH-H_2_O	>1,000	>1,000	>1,000	934 ± 262	>1,000
MH-E1-SF	187 ± 7.07	115 ± 31.6	125 ± 10.7	172 ± 12.0	134 ± 29.3
MH-SFE1b	340 ± 23.8	559 ± 80.5	313 ± 21.0	342 ± 52.1	358 ± 31.9
MH-SFE1c	182 ± 43.0	640 ± 173	262 ± 12.2	235 ± 15.1	150 ± 9.4
*Microalgae*					
GLE1	445 ± 22.9	>1,000	527 ± 3.9	465 ± 90.3	396 ± 27.3
GLE1-CF	322 ± 37.9	501 ± 58.6	281 ± 15.0	347 ± 53.2	246 ± 10.9
GLE1-CF-SF	199 ± 52.4	231 ± 13.2	108 ± 9.1	131 ± 16.6	131 ± 1.9

For the abbreviations of the extracts, please refer to Table 1. MCF-7, human breast epithelial adenocarcinoma; HepG2, human hepatocellular carcinoma; HOS, human osteosarcoma; D-17, canine osteosarcoma; and DH-82, canine histiocytosis.

**Table 4:** Combination index (CI) from isobologram analysis of selected extracts of *I. obliquus* and microalgae with various ratios.

Cells	Ratios of the two extracts				
	MH : GL (1 : 1)	MH : GL (2 : 1)	MH : GL (1 : 2)	MH : GL (4 : 1)	MH : GL (1 : 4)
MCF7	1.27	1.15	1.15	0.42	0.41
HepG2	0.76	0.76	0.78	0.43	0.40
HOS	1.27	0.95	0.99	0.42	0.46
D-17	1.17	0.84	0.76	0.47	0.47
DH-82	1.17	0.80	0.82	0.49	0.45

MH, MH-E1-SF (sugar-free ethanol extract of *I. obliquus*), GL, GLE1-CF-SF (chlorophyll- and sugar-free ethanol extract of microalgae mixture); MCF-7, human breast epithelial adenocarcinoma; HepG2, human hepatocellular carcinoma; HOS, human osteosarcoma; D-17, canine osteosarcoma; and DH-82, canine histiocytosis. CI < 1: synergism, CI = 1: additive effect, and CI > 1: antagonism.

## Data Availability

All the data are presented in this article.
